# Anxiety, Mental Stress, and Sudden Cardiac Arrest: Epidemiology, Possible Mechanisms and Future Research

**DOI:** 10.3389/fpsyt.2021.813518

**Published:** 2022-02-03

**Authors:** Neeltje M. Batelaan, Adrie Seldenrijk, Odile A. van den Heuvel, Anton J. L. M. van Balkom, Antonia Kaiser, Liesbeth Reneman, Hanno L. Tan

**Affiliations:** ^1^Department of Psychiatry, Amsterdam University Medical Center (UMC), Vrije Universiteit Amsterdam, Amsterdam, Netherlands; ^2^Amsterdam Public Health Research Institute, Amsterdam, Netherlands; ^3^Amsterdam Neuroscience Research Institute, Amsterdam, Netherlands; ^4^Department of Anatomy and Neuroscience, Amsterdam University Medical Center (UMC), Vrije Universiteit Amsterdam, Amsterdam, Netherlands; ^5^Department of Radiology and Nuclear Medicine, Amsterdam University Medical Center (UMC), University of Amsterdam, Amsterdam, Netherlands; ^6^Department of Psychiatry, Amsterdam University Medical Center (UMC), University of Amsterdam, Amsterdam, Netherlands; ^7^Department of Clinical and Experimental Cardiology, Amsterdam University Medical Center (UMC), University of Amsterdam, Amsterdam, Netherlands; ^8^Netherlands Heart Institute, Utrecht, Netherlands

**Keywords:** sudden cardiac arrest (SCA), sudden cardiac death (SCD), anxiety, mental stress, biological mechanism, predisposing and precipitating risk factors

## Abstract

Sudden cardiac arrest (SCA) is a leading cause of mortality and morbidity in affluent societies, which underscores the need to identify persons at risk. The etiology of SCA is however complex, with predisposing and precipitating factors interacting. Although anxiety and mental stress have been linked to SCA for decades, their precise role and impact remain unclear and the biological underpinnings are insufficiently understood. In this paper, we systematically reviewed various types of observational studies (total *n* = 20) examining the association between anxiety or mental stress and SCA. Multiple methodological considerations challenged the summarizing and interpretation of the findings. For anxiety, the overall picture suggests that it predisposes for SCA in physically healthy populations (unadjusted OR = 2.44; 95% CI: 1.06–5.59; *n* = 3). However, in populations at risk for SCA (*n* = 4), associations were heterogeneous but not significant. Anxiety may partly predispose to SCA by contributing to other risk factors such as cardiovascular disease and diabetes mellitus via mechanisms such as unhealthy lifestyle and metabolic abnormalities. Mental stress appears to precipitate SCA, presumably by more directly impacting on the cardiac ion channels that control the heart's electrical properties. This may lead to ventricular fibrillation, the arrhythmia that underlies SCA. To advance this field of research, experimental studies that unravel the underlying biological mechanisms are deemed important, and most easily designed for mental stress as a precipitating factor because of the short timeframe. These proof-of-concept studies should examine the whole pathway from the brain to the autonomic nervous system, and eventually to cardiac ion channels. Ultimately, such studies may facilitate the identification of persons at risk and the development of novel preventive strategies.

## Introduction

Sudden cardiac arrest (SCA) refers to the sudden, unexpected cessation of the heart's pump function as a result of cardiac arrhythmia, most often ventricular fibrillation (VF). In VF, the electrical activation which initiates the heart's contractile function is uncoordinated and too rapid (>300/min), leading to the abolishment of a coordinated contraction. Consequently, blood circulation stops and unless a normal coordinated heart rhythm is restored, death will set in within minutes, called sudden cardiac death (SCD) (1).

Regarding the etiology of SCA, the a priori risk for SCA is elevated by various predisposing factors, because these factors result in morphological changes (e.g., scar tissue) and/or functional changes in the heart (e.g., downregulation or changes in the functional properties of cardiac ion channels that control the heart's electrical properties). In addition, precipitating factors result in dysfunction of the cardiac ion channels, thereby triggering the occurrence of VF and SCA. When the a priori risk is high, a small precipitating factor will be sufficient to elicit SCA, whereas a larger trigger is required when the a priori risk is lower. Among the most prevalent predisposing factors are cardiovascular disease (CVD) and diabetes mellitus (2). For example, in adults, the incidence of SCA is 6.0 per 1,000 person-years in those with CVD, vs. 0.8 per 1,000 person-years without CVD (3). Similarly, SCD risk is elevated up to 2.7-fold in patients with diabetes (4).

A precipitating factor disrupts cardiac ion channel function. The autonomic nervous system (ANS) controls the functional properties of these ion channels (5). Sympathetic activation triggers a pathway which eventually results in changes in cardiac ion channel function, i.e., increased calcium influx which sets the stage for delayed afterdepolarizations that may trigger VF in vulnerable people and hence SCA. Parasympathetic activation causes the opposite effects, i.e., reduced cardiac excitability. For instance, activation of acetylcholine-sensitive potassium channels in atrial cardiomyocytes causes hyperpolarization of the resting membrane potential, thereby counteracting atrial depolarization. This property is being investigated as a novel drug target for the treatment of atrial fibrillation (6). External factors impacting the ANS may thus influence cardiac ion channels. One of the best-known external factors is physical stress, causing sympathetic activation during which adrenaline binding to its receptor on muscle cells triggers the pathway mentioned above.

SCA is a major public health problem, causing 20% of total mortality in industrialized societies (7, 8). SCA most often occurs in the community (out-of-hospital cardiac arrest) where rescuers are most often too late to arrive, resulting in low survival rates, ranging from 4 to 27% across Europe (9). Moreover, those who survive may suffer significant and persistent disabilities due to long-lasting hypoperfusion of the heart and the brain (7, 10). In upcoming years, the incidence and burden of SCA are expected to rise, because predisposing factors such as CVD and diabetes will become more prevalent in the aging world population. Insight in risk factors for SCA facilitates identification of persons at risk and, moreover, might contribute to understanding biological underpinnings, and thereby to the development of novel (secondary) preventive strategies.

For decades, anxiety symptoms and symptoms of mental stress have been linked to the occurrence of SCA (11). Whereas anxiety may be present without an external stressor, mental stress is regarded as a direct response to an external stressor (12). However, the distinction between these emotional responses is not always clear and symptom profiles are highly similar, including nervousness, difficulty sleeping and concentrating, fatigue, muscle tension, and irritability. The link between anxiety symptoms and symptoms of mental stress and SCA has been studied in various systematic reviews. For example, Strike and Steptoe concluded that mental stress caused by the experience of stressful public events such as earthquakes or emotionally challenging sporting occasions are probable triggers or precipitating factors for acute coronary syndromes, which in turn may lead to VF and subsequently to SCA and SCD (13). Likewise, in a previous meta-analysis, we have shown that anxiety symptoms are a predisposing factor for CVD mortality (including SCA), increasing the risk by 61% (14). However, these studies did not specifically examine SCA as the outcome measure. Overall, the possible role of mental stress or anxiety on the specific cardiac endpoint of SCA has received little attention.

As a result, the impact of anxiety or mental stress on SCA remains unclear. Moreover, the biological underpinnings are insufficiently understood. This article aims to expand on previous research by systematically searching the literature for studies examining the strength of the association between anxiety symptoms or mental stress symptoms and (non-)fatal SCA in the general population and patients with increased vulnerability for SCA. We thereby focus on studies with a long timeframe that are best suited to study predisposing risk factors, as well as on studies with a shorter timeframe which are more adequate to study direct triggers for SCA. In the discussion, we relate the findings concerning the predisposing and triggering role of anxiety/mental stress on SCA to the potential biological mechanisms and address the next steps in research. Ultimately, understanding whether and how anxiety and mental stress play a part in SCA may facilitate the development of (secondary) preventive strategies that are so dearly needed.

## Methods

To examine the association between anxiety or mental stress and SCA, a systematic search was performed in Embase, PubMed and APA PsycInfo of scientific literature in English dating up until May 21, 2021. Examining this association is complicated for various reasons, such as the difficulty to collect sufficient numbers of patients in whom SCA is ascertained, unclear biological mechanisms and consequently unclear timeframe, etc. We therefore aimed at gathering evidence from different angles, which has resulted in a broad search strategy. Eligible studies investigated the influence of anxiety or mental stress on the risk of SCA/SCD using a comparative, observational design. Studies on anxiety or mental stress were included independent of severity but had to use a psychiatric diagnosis of anxiety disorders according to the DSM, or an anxiety or mental stress questionnaire. Studies using proxies for experienced mental stress were also included, such as earthquakes or the presence of stressful life events. Since the biological underpinnings are insufficiently understood, and appropriate timeframes for predisposing or triggering effects could thus not be defined, studies were selected independent of timeframes used. We qualitatively analyzed unadjusted and multivariable-adjusted risk estimates [risk ratio (RR), odds ratio (OR), hazard ratio (HR), or absolute numbers with details of statistical tests, as reported by the original studies] to estimate the association between anxiety or mental stress and fatal or non-fatal SCA. If possible, we meta-analyzed unadjusted ORs and 95% CIs, using the “admetan” module in Stata 17 (15). As the incidence of SCA is relatively low [0.5–1/1,000/year (16)], ORs or HRs were assumed to be accurately close to estimates of the RR. ORs were calculated and forest plots are shown, using original data on dichotomized exposure and outcome data (2 × 2 tables), for both healthy and populations at risk. If anxiety/mental stress was classified into more than two categories, the categories with higher exposure were compared with the category with lowest exposure. Pooled ORs were calculated using random-effects models, assuming that the true effects would vary across studies. Potential heterogeneity among studies was calculated using the *I*^2^ statistic, which is a quantitative measure of inconsistency across studies. Studies were pooled irrespective of timeframe, considering the trade-off between inclusiveness vs. heterogeneity, and since the most appropriate timeframes to study predisposing or precipitating effects are unknown. See Supplementary Material 1 for detailed methods, including the search strategy and flow chart.

## Results

Our search identified 1,580 unique records. After title or abstract screening and full-text assessment, 20 studies were included in the systematic review. The publications were subdivided into three types of observational studies (Table 1). In type I, researchers directly examined the association between the presence or severity of anxiety and subsequent SCA or SCD (*n* = 7 studies) (17–23). In type II, researchers compared the rates of SCA or SCD during or directly after a public event likely to be accompanied by mental stress (Covid-19 pandemic, earthquakes, terrorist attack, important football tournaments) with those during control periods (*n* = 8 studies) (24–31). In type III, researchers retrospectively examined the life events in the period before SCA described by SCA survivors or relatives of SCA victims as compared with control persons or periods (*n* = 5 studies) (32–36). These three types of observational studies substantially differed in the timeframes examined. Longer time intervals are required to study anxiety/mental stress as a predisposing factor, and short time intervals are appropriate to study anxiety/mental stress as a triggering factor. In type I studies, SCA in the general population was examined over time intervals of 2 years (23), 12 years (20), and 32 years (22), whereas studies in populations at risk generally used a shorter time interval (1–3 years) (17–19, 21). By contrast, type II studies assessed SCA during or in the days or weeks following the emotionally charged event. Finally, type III studies used a timeframe of 1 month to up to 1 year prior to the SCA/SCD event. Of note, it appeared that studies with a longer timeframe examined anxiety symptoms (i.e., as predisposing factor), whereas studies with a shorter timeframe examined mental stress (i.e., as triggering factor).

**Table 1:** Summary of included studies.

	**References**	**Study design, sample *n***	**Anxiety or mental stress measure**	**Findings**
Type I	Li et al. (17)	Retrospective cohort with propensity score matching, *n* = 26,204	Pre-existing anxiety disorders	The incidence of in-hospital SCA was lower in the anxiety group than in the non-anxiety group of MI patients. This protective effect was only found in patients with non-ST segment elevation MI.
	Habibović et al. (18)	Prospective cohort, *n* = 1,012	STAI-S at time of ICD implantation	Anxiety increased the incidence of aborted SCD over 1-year follow-up. The associations remained significant after adjustment for multiple variables and were similar for men and women.
	Watkins et al. (19)	Prospective cohort, *n* = 947	CCEI phobic anxiety subscale	Overall, there was no association between anxiety and SCD. In women, however, anxiety increased the risk of SCD.
	Albert et al. (20)	Prospective cohort, *n* = 71,162	CCEI phobic anxiety subscale	Anxiety increased the risk of SCD in a sample of healthy women over a 32-year follow-up. The associations remained significant after adjustment for multiple variables but were attenuated after further adjustment for various comorbidities.
	Frasure-Smith et al. (21)	Prospective cohort, *n* = 222	STAI-S at time of post-MI hospital admission	Anxiety was not significantly associated with incident fatal or non-fatal SCA in the first year post-MI.
	Kawachi et al. (22)	Case-control in a prospective cohort, *n* = 1,895	Anxiety symptom scale out of 5 questions from the CMI	Anxiety increased the risk of SCD in a sample of healthy men over a 12-year follow-up, also after adjustment for several variables.
	Kawachi et al. (23)	Prospective cohort, *n* = 33,858	CCEI phobic anxiety subscale	Anxiety increased the risk of SCD in a sample of healthy men over a 2-year follow-up, also after adjustment for several variables.
Type II	Marijon et al. (24)	Cross-sectional database	Paris COVID-19 lockdown vs. control period	During the Paris lockdown, the maximal weekly SCA incidence was significantly higher than during control periods, and a rapid return to normal was seen in the final weeks.
	Simon et al. (25)	Cross-sectional database	Football tournaments (2012, 2016, 2018) vs. control periods.	There was no increased SCA incidence during the (combined) football tournament periods vs. control periods in Polish men.
	Niiyama et al. (26)	Cross-sectional database	Great East Japan earthquake and tsunami 2011 vs. control periods	For the initial 4 weeks after the earthquake, the incidence of SCD in the Iwate prefecture was doubled as compared with control periods, after which it returned to baseline.
	Niederseer et al. (27)	Cross-sectional database	Football tournament days the German team played (2006) vs. control periods	There was no increased incidence of SCA hospital admissions during the football tournament vs. control periods in men and women in the German province of Bavaria.
	Aoki et al. (28)	Cross-sectional database	Great East Japan earthquake 2011 vs. control periods	The number of SCA ambulance transports was significantly increased in the week following the earthquake as compared with control periods; it peaked on day 2 followed by a gradual decline.
	Gold et al. (29)	Cross-sectional database	Nisqually earthquake 2001 and 9-11 terrorist attack 2001 (USA) vs. control periods	The incidence of SCD increased during the 48 h following the Nisqually earthquake, as compared with control periods. No increased SCD incidence was found during 1-week post-earthquake, nor during 48-h or 1 week after the 9-11 terrorist attack.
	Katz et al. (30)	Cross-sectional database	FIFA Football World Cup 2002 vs. control period	The incidence of SCD increased during the FIFA in both men and women.
	Leor et al. (31)	Cross-sectional database	Northridge (USA) earthquake 1994 vs. control periods	The incidence of SCD increased on the day of the Northridge earthquake as compared with control periods and afterwards returned to baseline level.
Type III	Chang Liu et al. (32)	Case-control, *n* = 72	LCUs over 1 year before SCA, based on the SRSS, SSS, or RLCQ	SCA survivors did not significantly differ from matched controls with respect to stressful life events during the year before the SCA.
	Jeong et al. (33)	Case-control, *n* = 190	Major life events in 1 year before SCA or control periods based on family	SCA survivors had a higher number of major life events than matched controls.
	Wicks et al. (34)	Case- crossover, *n* = 490	Major life events in 1 vs. 2–6 months before SCA, based on spouse interviews.	Major life events occurred more frequently during the 1 month before the SCA than during the control period.
	Cottington et al. (35)	Case-control, *n* = 162	Major life events in 6 months before SCD, based on interviews with spouses or next of kin.	SCD cases had experienced a similar number of life events (total, negative, unclassifiable) during 6 months before death as had neighborhood controls, however, they had experienced less positive life events and more often the death of a significant other.
	Rahe and Lind (36)	Case- crossover, *n* = 39	LCUs over 3 years before SCD based on the Schedule of Recent Events questionnaire by spouses or next of kin.	LCUs were higher in the 6 months before SCD than during the control periods in both healthy subjects and those at risk (CHD history).

*CCEI, Crown-Crisp experiential index; CHD, coronary heart disease; CMI, Cornell Medical index; ICD, implantable cardioverter defibrillator; LCUs, life change units. LVEF, left ventricular ejection fraction; MI, myocardial infarction; SCA, sudden cardiac arrest; SCD, sudden cardiac death; SRRS, Social Readjustment Rating Scale; SSS=Student Stress Scale; STAI-S, state portion of Spielberger's State-Trait Anxiety Inventory; RLCQ, Recent Life Changes Questionnaire*.

### Type I: Anxiety and Fatal or Non-fatal SCA

Seven studies examined the association between anxiety and SCA or SCD during follow-up, five of which used a prospective cohort design (18–21, 23), one used a retrospective cohort (17), and one a case-control (22) design. In one study, the exposure was based on pre-existing anxiety disorders (17), whereas most studies used severity of anxiety symptoms, i.e., the state portion of Spielberger's State-Trait Anxiety Inventory (STAI-S) (18, 21), the phobic anxiety subscale of the Crown-Crisp experiential index (CCEI) (19, 20, 23), or anxiety symptoms based on the Cornell Medical Index (CMI) (22). Follow-up duration ranged from 1 to 32 years. Only three studies were based on participants free from CVD at baseline (20, 22, 23), whereas the remaining studies included patients who already had increased vulnerability for SCA. For more details on the individual studies, see Supplementary Material 2.

#### Physically Healthy Populations

**Figure 1:**
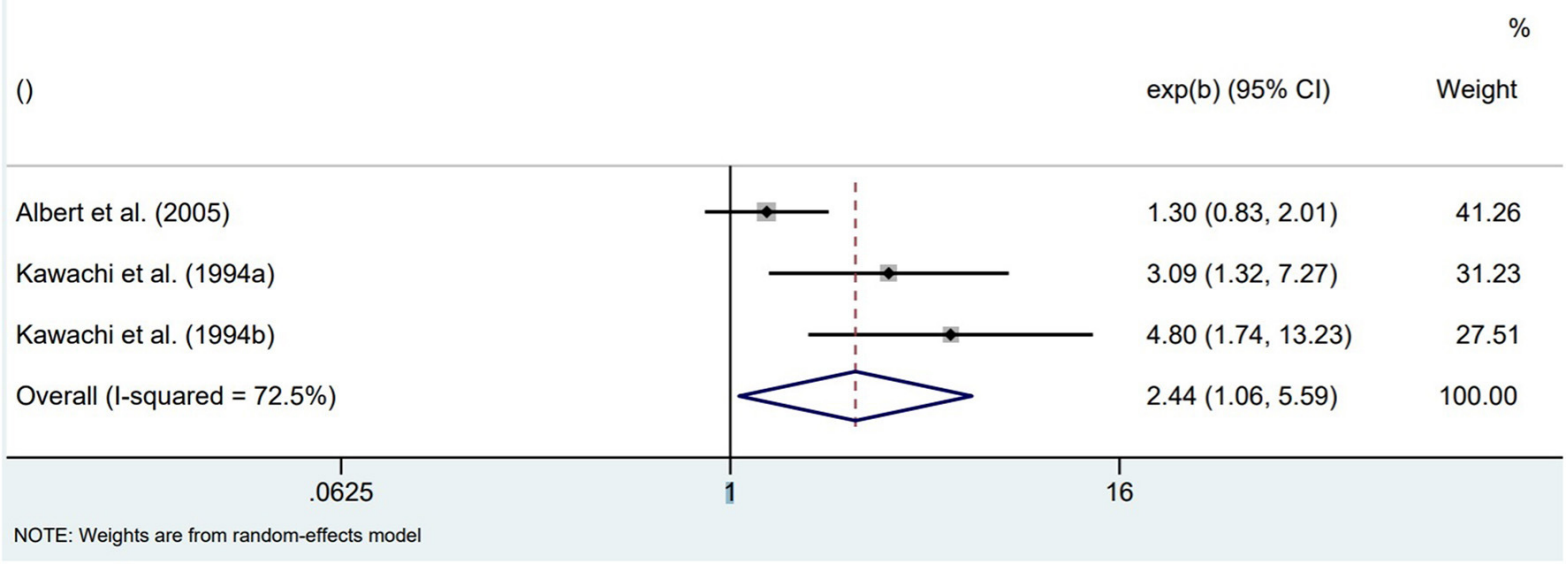
Pooled unadjusted effect measures of studies on anxiety symptoms and SCD in physically healthy individuals.

All prospective cohort studies in physically healthy participants found an increased risk for SCD in those participants who were more anxious at baseline. Based on the Nurses' Health Study data of 71,162 registered female nurses without CVD or cancer at baseline, both continuous (p-trend across quartiles of CCEI phobic anxiety) and dichotomized (highest vs. lowest CCEI quartile) anxiety symptoms at baseline were associated with SCD over the 12 years of follow-up (20). Associations remained statistically significant when adjusted for age and risk factors of coronary heart disease (CHD) but were attenuated after further adjustment for various comorbidities (hypertension, diabetes, hypercholesterolemia). Based on the Normative Aging Study data of 1,895 community-dwelling male veterans without chronic medical conditions at baseline, higher anxiety at baseline (CMI scores ≥1) was associated with increased odds of SCD over 32 years of follow-up, also after adjustment for CHD risk factors, although the small numbers resulted in imprecise risk estimations (SCD events per CMI category: 19/1,670, 5/171, 2/35) (22). Based on the Health Professionals Follow-up Study data of 33,858 male health professionals free of diagnosed CVD at baseline, higher anxiety at baseline (CCEI scores ≥ 2) was associated with increased odds of SCD over 2 years of follow-up, also after adjustment for CHD risk factors (significant p-trend across CCEI categories; the small numbers resulted in imprecise risk estimations of SCD events per CMI category: 5/23,259, 2/4,821, 7/3,485, 2/2,293) (23). Pooling the unadjusted associations between dichotomized anxiety symptoms at baseline (higher categories vs. lowest category) and incidences of SCD at follow-up in initially physically healthy populations resulted in significantly increased odds of SCD for highly anxious individuals (OR = 2.44, 95%CI 1.06–5.59; Figure 1).

#### Populations at Elevated Risk for SCA

**Figure 2:**
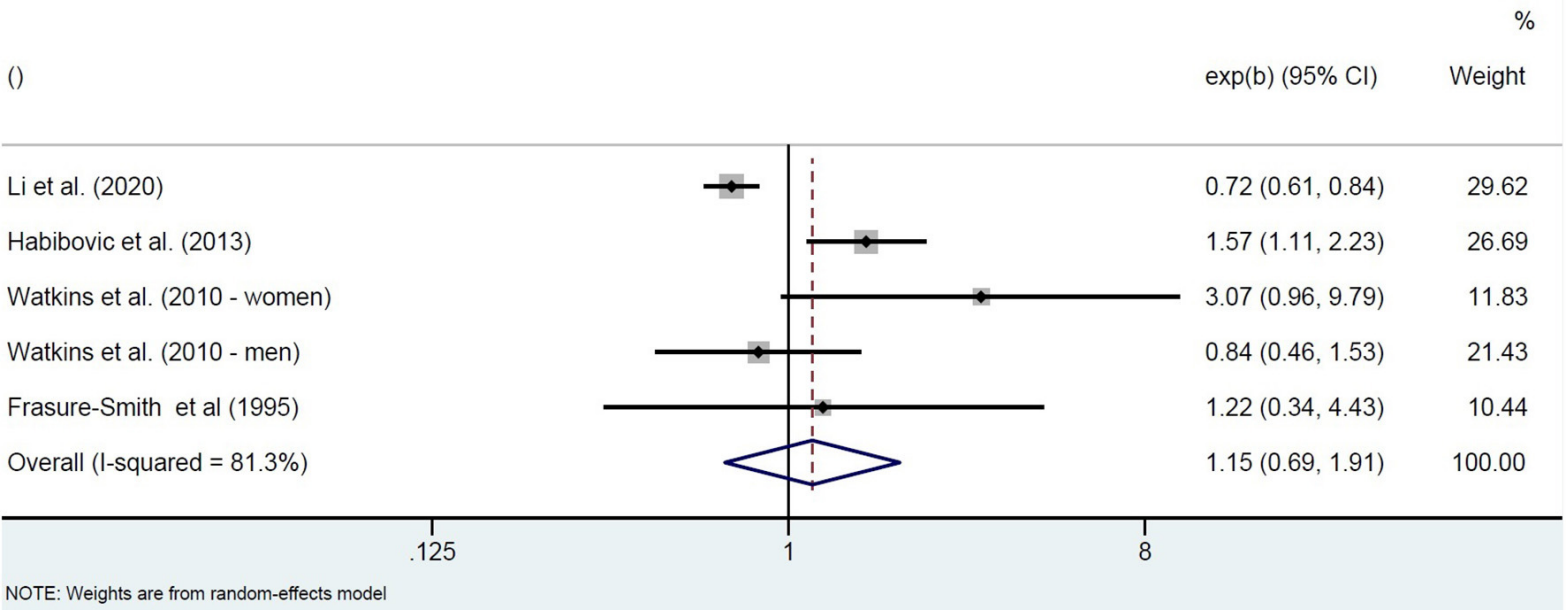
Pooled unadjusted effect measures of studies on anxiety and SCA in individuals with elevated SCA risk.

The four included studies investigating populations at elevated risk for SCA show highly heterogeneous results. One prospective cohort study in patients with implantable cardioverter defibrillators (ICDs) found that patients with higher anxiety symptoms at ICD insertion (STAI-S) had a higher 1-year incidence of ventricular arrhythmias, also after adjustment for covariates (18). Two prospective cohort studies in patients with an acute MI or with angina admitted for diagnostic cardiac catheterization found that high anxiety at baseline was not associated with fatal or non-fatal SCA. High anxiety was defined as a STAI-S score of at least 40 measured 5–15 days post-MI (21), or as the highest quartile of the CCEI phobic anxiety scale measured 0-4 days post-catheterization (19). One of these studies (VAGUS cohort) also showed that, in contrast to men, highly-anxious women had a significantly increased risk of SCD over 3 years of follow-up (19). In the other study, however, it was mentioned that all cases of arrhythmic events over the 1-year follow-up period occurred in men (21). Finally, a large retrospective cohort using the 2016 National Inpatient Sample data of MI patients showed a protective effect of pre-existing anxiety disorders on the incidence of in-hospital SCA, but only in non-ST segment elevation MI patients (17). Pooling the unadjusted associations of these studies between baseline anxiety and incidence of SCA resulted in highly heterogeneous effect estimates, with the pooled odds for higher symptoms of anxiety not being significantly different from low symptoms of anxiety (OR = 1.15, 95%CI 0.69–1.91; Figure 2).

### Type II: Emotionally Charged Public Events and Fatal or Non-fatal SCA

Eight studies compared the rates of SCA or SCD during or directly after an emotionally challenging public event (Covid-19 pandemic, earthquakes, terrorist attack, important football tournaments as proxies for mental stress) with those during control periods (24–31). These “natural experiment” studies are based on records of insurance companies or the authorities of a certain catchment area. Due to the large heterogeneity in effect measures and standardization, results of type II studies have not been pooled. For more details of the individual studies, see Supplementary Material 3.

#### Humanitarian Disasters

Studies on the effects of four out of five selected humanitarian disasters were suggestive of a relative increase in (non)fatal SCA rates. For instance, the weekly incidence of SCA during the Paris COVID-19 lockdown was significantly higher than during control periods (24). Likewise, data on the Northridge earthquake 1994 and the Nisqually earthquake 2001 showed increased SCD rates in the affected countries as compared with the control periods (29, 31). Furthermore, data on the Great East Japan earthquake and tsunami 2011 also showed increased SCD or SCA rates in affected prefectures (26, 28). With respect to the timeframes, three of the earthquake studies reported that the incidence of (non)fatal SCA was particularly increased on either the day of or the day after the event (28, 29, 31). The 9–11 terrorist attack in 2001, however, gave no rise to SCD rates in King County (situated far away from the place of the terrorist attacks) (29).

#### Football Matches

The three studies on the effects of football tournament periods were less suggestive of an association between mental stress and (non)fatal SCA. Only one study found a significantly increased incidence of SCD in Swiss men and women during the FIFA World Cup 2002 as compared with the control period (30). Two other studies, covering four tournament periods, found no significant associations between football spectatorship and the incidence of SCA in Polish men (25) or German men and women in the province of Bavaria (27).

### Type III: Stressful Life Events and Fatal or Non-fatal SCA

Five studies examined the experienced recent life events (as proxy for mental stress) described by SCA survivors or relatives of SCA victims as compared with control persons or periods (32–36). For more details of the individual studies, see Supplementary Material 4. Three studies based on information of spouses or relatives found an association between major life events and fatal or non-fatal SCA. One case-crossover study showed that the total stress-generating potential of experienced life events (life change units, i.e., counting life-events and weighting these for stress-generating potential) in the 6-months prior to SCD in Swedish men was higher compared to the same period in two preceding control years (36). Likewise, another case-crossover study showed that the occurrence of a major life event involving family or friends was higher in the month before the SCA than in the control period of 2–6 months before the SCA (34). A case-control study also showed that SCA survivors had experienced a significantly higher number of major life events in the year prior to the SCA than had matched controls (33). In contrast, two other case-control studies found no association between life events and (non)fatal SCA. One study in a population with a broad age range found no significant differences between the SCA survivor group and matched controls with respect to the total stress-generating potential of experienced life events over the year prior to the SCA (32). Another study found that American women with SCD overall did not differ from the race, sex, and age-matched controls with respect to the total number of stressful life events in the 6 months prior to SCD, although they had experienced fewer positive life events and more often the death of a significant other (35).

## Discussion

### Main Findings

We may conclude from this systematic literature research that the evidence for the relationship between anxiety or mental stress and SCA or SCD derived from various types of studies is mixed.

#### Anxiety

The pooled results of three prospective cohort studies (type I) in healthy populations suggest that people with high anxiety scores had a more than 2-fold higher chance to experience SCA (20, 22, 23). This suggests that the risk of SCA in people with more severe anxiety symptoms is of similar magnitude as the risk associated with established predisposing risk factors such as diabetes (5). However, pooling four studies (type I) in populations at risk for SCA resulted in a non-significant effect estimate. It should be noted that in both meta-analyses heterogeneity was substantial (*I*^2^ higher than 70%).

Studies in healthy populations and in populations at risk both examined the effect of presence and severity of anxiety symptoms as a predisposing risk factor, but populations at risk in general had much shorter time intervals for follow-up measurements. It might be that anxiety symptoms indeed predispose to SCA by a process that requires time, which is thus better captured in studies with longer follow-up intervals. It might also be that the role of anxiety is primarily to contribute to a predisposition, and once the predisposition is present, its role is limited.

#### Mental Stress

Studies on the precipitating effect of symptoms of mental stress and SCA showed a mixed picture. Stress was only assessed indirectly by proxies, including natural disasters, football spectatorship, or by the experience of life events. It is likely that these sources of stress highly differ in intensity and in impact on disrupting life, which might explain the mixed results. Indeed, as would be expected, in type II studies, the evidence for an association with SCA was stronger for studies investigating natural disasters (particularly earthquakes) than for those on football spectatorship. Of the retrospective case-control and case-crossover studies on life events (type III studies), three studies found an association with SCA, whereas two studies did not.

### Methodological Considerations

The main strength of this systematic review is its broad scope, including the overlapping aspects of both anxiety and mental stress, both physically healthy and at-risk populations, and varying timeframes. In addition, we included various types of observational studies, each with its own assets, for example the possibility of adjustment for covariates in type I, the large sample size in type II, and the detailed assessment of the period prior to the SCA in type III. This broad scope was deemed necessary to study a complex association of which so little is known. By focusing on SCA specifically as outcome, we addressed limitations of previous research on the cardiac risk associated with anxiety and mental stress. However, per definition, examinations of outcomes that are assumed to have a multifactorial etiology such as SCA will often be surrounded by uncertainties, and more so when outcomes are less frequent. On top of that, several limitations should be mentioned. First, the timeframe chosen in studies is highly relevant. In general, to study predisposing factors, longer follow-up periods are required as compared to precipitating factors directly triggering SCA. More specifically, the most appropriate timeframe depends on the assumed underlying biological mechanisms. Any mismatch between the follow-up period and the time required for the biological mechanism to act will have an impact on findings, i.e., too long follow-up periods will dilute associations, whereas too short follow-up periods will not show effects. If more studies become available, it would be preferable to pool those with similar timeframes, which also could diminish the substantial heterogeneity found in the meta-analyses.

Second, in all three types of studies presented here, confounding factors may be substantial and are often not accounted for sufficiently. For example, increased rates of SCA during the COVID-19 pandemic might be due to fear and psychological stress due to the pandemic, but might also be due to other factors, such as COVID-19-related complications, temporarily limited access to the health care system (24, 37), or lifestyle changes during lockdown. Likewise, although persons with anxiety may use selective serotonin reuptake inhibitors (SSRIs), some of which have proarrhythmic effects (38), analyses were not adjusted for medication use. Moreover, only baseline assessments of time-dependent confounding variables (such as cardiovascular status) were used in the analyses. An individual patient data meta-analysis would shed the clearest light on the influence of (time-dependent) confounding factors at an individual level.

Third, the assessment of anxiety and mental stress is suboptimal. It appeared that anxiety was examined only as a predisposing factor precluding evidence for its potential triggering role. Likewise, mental stress only was examined as a triggering factor. The predisposing potential of (chronic) mental stress therefore remains unknown. Furthermore, in type I studies, the assessment of anxiety took place only once, at baseline, thereby neglecting the fact that anxiety may vary over time, especially over periods of multiple years. In both cases of anxiety symptoms either disappearing or developing after baseline, the effect estimates will have been biased toward zero. In addition, most type I studies assessed anxiety symptoms rather than disorders and the proportion of “clinically significant anxiety” (39, 40) was limited. As some studies reported a dose-response relationship, it is possible that assessing clinically significant anxiety only would have resulted in higher effect sizes. In type II and type III studies, mental stress was assessed indirectly by means of experienced life events, disasters, or emotionally challenging public events. In other words: no rating scale on presence or severity of stress was used. Such indirect assessments have various limitations. For example, we do not know exactly which emotions are triggered. We pragmatically labeled them as “mental stress,” thereby ignoring that, while positive life events may also trigger positive emotions, negative life events may trigger anxiety or mourning, and football matches may trigger emotions depending on the outcome of the match (41). Thus, “mental stress” is likely to encompass various emotions which might have a differential impact on the development of SCA. Moreover, measuring events rather than the emotions themselves assumes that these events are emotionally challenging for all people. This assumption is not likely to be true. For example, people not interested in football will not experience any emotions, thereby introducing a bias toward zero. Also, stress due to the 9–11 terrorist attacks is likely to be higher in places near the terrorist attacks than in distant places. Furthermore, the impact of unforeseeable events (e.g., earthquakes) might differ from that of foreseeable events (e.g., football matches).

Finally, retrospective assessments are prone to bias inflating effects, i.e., people and relatives are more prone to remember life events when followed by SCA in an attempt to understand the occurrence of the cardiac event (42). Finally, data obtained from relatives is indirect, thereby hampering reliability.

In sum, the findings of the present meta-analysis and systematic review are surrounded by uncertainties due to methodological aspects. Overall, the estimated risk for SCA associated with anxiety/mental stress is modest, suggesting that other factors play a role and may even modify the effect of anxiety and mental stress.

### Potential Biological Pathways

#### Predisposing to SCA

Findings of type I studies that were conducted in the general population and mainly had long follow-up intervals suggest that anxiety symptoms increase the risk for SCA by more than 2-fold. Anxiety thus seems to predispose individuals to SCA by processes requiring time. This may also explain why findings in populations at risk for SCA were less clear, i.e., follow-up periods were restricted to 1 or at most 3 years which may be short. In the general population studies, the risk estimate of anxiety decreased when analyses were adjusted for known risk factors for SCA such as CVD and diabetes. Hence, the presence of anxiety symptoms partly increases the a priori risk for SCA by associations with CVD and diabetes. Indeed, we previously reported that anxiety symptoms are associated with the onset of CVD (14). The underlying biological mechanisms may range from the association of anxiety disorders with unhealthy lifestyle factors (i.e., smoking, lower physical activity, and poor diet) to metabolic abnormalities (i.e., hypercholesterolemia, immune dysregulations, hypercoagulability). These may promote atherosclerosis and subsequently result in the onset of CVD (14). Likewise, anxiety is associated with the onset of diabetes mellitus (43). Though the biological underpinnings of this association need to be unraveled, inflammation and also cardiometabolic abnormalities might play a role (43).

When analyses were adjusted for multiple lifestyle and biological measures, the risk remained present. This suggests that anxiety symptoms not only impact SCA via their influence on other predisposing factors mentioned above. The increased risk estimate might be due to other, yet unknown, biological pathways which also require time. Moreover, as anxiety often runs a chronic course, it may also be that anxiety at baseline is associated with (peaks of) anxiety years later, and that such later (peaks of) anxiety trigger the direct onset of SCA.

#### Precipitating SCA

In most type II studies, emotionally challenging humanitarian disasters led to higher rates of SCA, and the risk was highest directly following the disaster, returning to normal levels fast (24, 26, 28, 29, 31). This suggests that stressful or emotional situations may acutely elicit SCA when stress levels are highest. Likewise, we have found the risk was less clear or absent in football matches, or for disasters happening relatively far away (29), suggesting that the amount of mental stress impacts on SCA, which is in line with Hill's criterion of biological gradient (44). Type III studies, examining the impact of life events on SCA, used a timeframe of 1 month until 1 year prior to the SCA/SCD event. Due to this design, the time relationship between the highest stress level and occurrence of SCA is less clear in these studies.

It is hypothesized that the underlying biological mechanism by which mental stress elicits SCA involves cardiac ion channels, as these ion channels directly control the heart's electrical properties. The normal cardiac cycle is composed of a depolarization (activation) phase, a repolarization phase, and a resting phase between heartbeats. Ion channel dysfunction causing VF may occur in sodium and calcium ion channels important in the depolarization phase, or in various potassium channels, important in the repolarization phase. The functional properties of these ion channels are controlled by a balanced system of sympathetic and parasympathetic regulators of the ANS (5), which respond in turn to the central control of the brain. In various inherited or common acquired conditions, sympathetic stimulation is associated with cardiac arrhythmias underlying SCA. The inherited arrhythmia syndromes most strongly associated with mental stress-induced arrhythmias and SCA are long QT syndrome type 2 (LQTS2) (45), catecholaminergic ventricular tachycardia (CPVT) (46) and an idiopathic VF syndrome linked to a risk haplotype that contains the DPP6 gene (5). Arrhythmogenic right ventricular dysplasia/cardiomyopathy (47, 48) and LQTS type 1 (45) are also strongly triggered by sympathetic stimulation, particularly following physical stress. In common acquired conditions, the clearest example of cardiac arrhythmia evoked by mental stress-induced sympathetic stimulation is electrical storm. Following acute management of this cardiac emergency (49), psychiatric treatment may play a role in its long-term treatment (50).

Dysfunction of ion channels due to mental stress may primarily occur during the cardiac repolarization phase. This notion derives from documentation of changes in the ST-T segment of the electrocardiogram (ECG) during mental stress (51–53), reflecting changes in cardiac repolarization. Moreover, LQTS2 predisposes to SCA by gene mutations in the KCNH2 gene, which encodes the potassium ion channels at play during repolarization (52).

### Next Steps in Research

Our review of observational studies underpins the idea that anxiety and mental stress influence the occurrence of SCA. However, due to the multiple methodological limitations of observational studies as mentioned above, the causality of this relationship will be difficult to prove, if not impossible. This hampers the development of preventive strategies. To advance research on the cardiac effects of stress, experimental studies unraveling the underlying biological mechanisms are required. Due to the short timeframe, such studies are easier to design for precipitating factors as opposed to predisposing factors. Findings from these studies might support the causality criteria of plausibility and coherence as formulated by Hill (44).

So far, most studies have focused on peripheral effects, i.e., effects of neurotransmitters (mostly catecholamines) on the heart and its constituent ion channels. Therapies based on these insights have limited applicability, either because they are only partly effective in SCD prevention (e.g., β-adrenoceptor blockers), or because they involve a high risk for side-effects and are therefore used in highly selected patients only (e.g., stellectomy). Emerging evidence suggests a role for central control of cardiac function by specific brain regions, such as the ventromedial prefrontal cortex, insular cortex, amygdala, bed nucleus of the stria terminalis, hypothalamus and periaqueductal gray (54, 55).

In our opinion, the scope of experimental studies to examine the precipitating role of mental stress for SCA should therefore not be limited to peripheral effects. Rather, proof-of-concept studies should be extended to the whole pathway from the brain to the cardiac ion channel function. The amygdala should be of particular interest, since it is crucial in processing mental stress, and its activity has already been related to the occurrence of cardiovascular events (56). The hypothalamus-pituitary-adrenal (HPA) axis projects onto the hypothalamus through the most prevalent inhibitory (gamma-amino butyric acid; GABA) and excitatory (glutamate) neurotransmitters in the hippocampus, amygdala and prefrontal cortex (57, 58). Moreover, rat studies found a significant imbalance between GABA and glutamate after exposure to acute and chronic stress (59, 60). Not many studies have investigated the influence of acute stress on the human glutamate and GABA concentrations. One magnetic resonance spectroscopy (MRS) study investigating stress using a psychosocial stressor found no changes in the prefrontal cortex (61). In contrast, two other MRS studies reported imbalance between GABA and glutamate after chemically induced panic or threat of shock, either by increased glutamate (62) or decreased GABA (63) concentrations in the prefrontal cortex. Results on metabolite changes induced by acute stress are still scarce, but it has been suggested that the direction of GABA concentration changes after acute stress is stressor dependent, both in the hippocampus (64) and in the frontal cortex (65, 66).

The priority therefore lies with studies investigating whether (different kind of) stress-induced changes in brain functioning and alterations in GABA and glutamate are indeed associated with changes in ECG markers of SCA risk. Consequently, if mental stress indeed reduces GABA concentrations in the amygdala and changes cardiac electrophysiology, future studies should investigate whether an altered SCA risk is associated with changes in GABA-ergic metabolism based on medication use or a particular genetic profile. Based on these findings, pharmacological treatment and development could then be advanced to reduce SCA risk (i.e., GABA-ergic pharmacotherapy of cardiac arrhythmias). In view of the addictive properties of most GABA-ergic drugs, we would also like to advocate more studies into the efficacy of behavioral therapy to prevent cardiac arrhythmias. This could involve learning a more adaptive stress response using functional Magnetic Resonance Imaging (fMRI) neurofeedback or cardiac physiology output-based training of bodily stress regulation (biofeedback).

Thus, studying stress-induced changes in the brain and cardiac electrophysiology will help to unravel the underlying biological mechanisms by which stress increases SCA risk. These insights may be used to identify individuals at risk and to develop preventive interventions to reduce SCA rates. For example, structural or functional changes in particular brain regions or certain molecular signaling pathways could serve as a biomarker. Likewise, these identified molecular signaling pathways could aid the development of novel (drug) treatments.

## Conclusion

By summarizing observational studies this review supports the prevailing idea that anxiety and mental stress may increase SCA risk. Anxiety predisposes (particularly physically healthy persons) to SCA by processes requiring time, partly by contributing to other risk factors for SCA. Mental stress may elicit SCA, presumably by impacting upon cardiac ion channels. To advance the field, experimental studies examining the precipitating role of mental stress for SCA are required to complement observational studies. These should encompass the whole pathway from the brain to cardiac ion channel dysfunction.

## Author Contributions

NB, OH, LR, and HT: study design. AS and AB: data collection and analysis. All authors: interpretation of findings, drafting manuscript, critical revision, and approval of final version of the manuscript.

## Funding

HT had received funding from the European Union's Horizon 2020 research and innovation program under acronym ESCAPE-NET, registered under grant agreement No 733381. The ESCAPE-NET project's primary aim is to better understand the causes of sudden cardiac arrest, including the mechanisms by which mental and physical stress trigger the cardiac arrhythmias that underlie sudden cardiac arrest. The funder had no role in the design, data collection, data analysis, data interpretation or writing of the report.

## Conflict of Interest

The authors declare that the research was conducted in the absence of any commercial or financial relationships that could be construed as a potential conflict of interest.

## Publisher's Note

All claims expressed in this article are solely those of the authors and do not necessarily represent those of their affiliated organizations, or those of the publisher, the editors and the reviewers. Any product that may be evaluated in this article, or claim that may be made by its manufacturer, is not guaranteed or endorsed by the publisher.
