# Supplementary material for: Anxiety, Mental Stress, and Sudden Cardiac Arrest: Epidemiology, Possible Mechanisms and Future Research
Source: Front Psychiatry. 2022 Feb 3;12:813518. doi: 10.3389/fpsyt.2021.813518 (PMC8850954; doi:10.3389/fpsyt.2021.813518)
Supplement: Supplementary file 1 [file Data_Sheet_1.docx]

**Supplement 1. Methods**

*Search*

A search was performed in Embase, PubMed and APA Psychinfo of scientific literature dating up till May 21, 2021. We also searched reference lists of pertinent review articles to gain access to additional literature (see **Figure 1. Flow chart**).

Records identified through database searching (n=1986)

Additional records identified through other sources (n=22)

Records screened on title and abstract (n = 1592)

Full-text articles assessed

for eligibility (n = 49)

Full-text articles excluded with reason (n=29)

- design, n = 8
- outcome, n = 17
- predictor, n = 4

Studies included (n = 20)

Records excluded after screening (n=1543)

Records after duplicates removed

(n = 1592)

***Supplemental Figure 1. Flowchart presenting the literature search strategy and results***

*Selection and data extraction*

The following pre-specified inclusion criteria were used: written in English, comparative, observational design, investigating the influence of anxiety or emotional distress on the risk of fatal/non-fatal sudden cardiac arrest (SCA). Studies on aborted SCA were included, i.e. ventricular arrhythmias in patients with an Implantable Cardioverter Defibrillator. Anxiety or mental distress had to be based on a psychiatric diagnosis of anxiety disorders according to the DSM, an anxiety or life stress questionnaire, or a list of stressful events used in an interview. Studies using proxies for experienced emotional distress were also included, e.g. earthquakes, spectatorship at important football games, etc. Case-control studies with an active control group (e.g. other heart condition than SCA) were excluded, as were studies with combined outcomes (e.g. myocardial infarction or SCA) in which SCA was not separately reported. The selection procedure was performed independently by two of the authors [AS and AJLMvB] by using Rayyan Systems Inc.[1] and inconsistencies in results were discussed and resolved before proceeding, if needed with help of a third author [HLT]. The same couple of authors [AS, AJLMvB] also extracted the following data from eligible studies: study design, dataset, population (age, sex,), anxiety or mental stress measure, cardiac arrest outcome (SCA including aborted SCA, or sudden cardiac death), main findings.

[1] Mourad Ouzzani, Hossam Hammady, Zbys Fedorowicz, and Ahmed Elmagarmid. Rayyan — a web and mobile app for systematic reviews. Systematic Reviews (2016) 5:210, DOI: 10.1186/s13643-016-0384-4.

*Search strategy databases*

**PubMed search 21-5-2021**

|  | Query | Results |
| --- | --- | --- |
| #8 | Search: #4 OR #7 Sort by: Most Recent | 447 |
| #7 | Search: #6 AND #2 Sort by: Most Recent | 124 |
| #6 | Search: "sudden cardiac death*"[ti] OR "sudden cardiac death*"[ot] OR "sudden cardiac arrest*"[ti] OR "sudden cardiac arrest*"[ot] Sort by: Most Recent | 7,185 |
| #4 | Search: #1 AND #2 AND #3 Sort by: Most Recent | 351 |
| #3 | Search: "Epidemiologic Studies"[Mesh] OR Cohort[TIAB] OR longitudinal[TIAB] OR prospective*[TIAB] OR "follow-up stud*"[tiab] OR "case-control stud*"[tiab] OR "retrospective stud*"[tiab] OR "cross-sectional stud*"[tiab] Sort by: Most Recent | 3,399,481 |
| #2 | Search: "Stress, Psychological"[Mesh] OR "Trauma and Stressor Related Disorders"[Mesh] OR "Anxiety" [Mesh] OR "Anxiety disorders"[Mesh] OR anxiet*[tiab] OR anxious*[tiab] OR worry* [tiab] OR worries* [tiab] OR Panic* [tiab] OR phobi* [tiab] OR "post-traumatic stress"[tiab] OR "posttraumatic stress*"[tiab] OR agoraphobi*[tiab] OR "obsessive compulsive disorder*"[tiab] OR OCD[tiab] OR "psychological stress*"[tiab] OR "psychological distress*"[tiab] OR "emotional stress*"[tiab] OR "emotional distress*"[tiab] OR psychotrauma*[tiab] OR "Mental stress*"[tiab] OR "mental distress*"[tiab] OR PTSD[tiab] OR "traumatic stress*"[tiab] OR "traumatic distress*"[tiab] OR burnout*[tiab] OR "burn-out*"[tiab] OR "work stress*"[tiab] OR "occupational stress*"[tiab] OR "compassion fatigue*"[tiab] Sort by: Most Recent | 494,833 |
| #1 | Search: "Heart Arrest"[Mesh] OR "Arrhythmias, Cardiac/mortality"[Mesh] OR "sudden cardiac arrest*"[tiab] OR "sudden cardiac death*"[tiab] OR "Karoshi Death*"[tiab] OR "cardiac death"[tiab] OR "cardiac mortalit*"[tiab] OR "sudden death*"[tiab] OR "heart arrest*"[tiab] OR "heart arrhythmia*"[tiab] OR "heart ventricular arrhythmia*"[tiab] OR "heart ventricular tachycardia*"[tiab] OR "sudden heart death*"[tiab] OR "cardiac arrest*"[tiab] Sort by: Most Recent | 110,212 |

**Embase search 21-5-2021**

| **No.** | **Query** | **Results** |
| --- | --- | --- |
| **#8** | **#7** AND (**'article'**/it OR **'article in press'**/it OR **'conference abstract'**/it OR **'conference paper'**/it OR **'conference review'**/it OR **'review'**/it OR **'short survey'**/it) | **1,038** |
| **#7** | **#5** OR **#6** | **1,088** |
| **#6** | **#2** AND **#3** | **252** |
| **#5** | **#1** AND **#3** AND **#4** | **897** |
| **#4** | **'case control study'**/de OR **'cohort analysis'**/de OR **'cross sectional study'**/de OR **'longitudinal study'**/de OR **'prospective study'**/de OR **'retrospective study'**/de OR **'follow up'**/exp OR **cohort**:ab,ti,kw OR **longitudinal**:ab,ti,kw OR **prospective***:ab,ti,kw OR **'follow-up stud*'**:ab,ti,kw OR **'case-control stud*'**:ab,ti,kw OR **'retrospective stud*'**:ab,ti,kw OR **'cross-sectional stud*'**:ab,ti,kw | **4,804,062** |
| **#3** | **'mental stress'**/exp OR **'anxiety disorder'**/exp OR **'anxiety'**/exp OR **anxiet***:ab,ti,kw OR **anxious***:ab,ti,kw OR **worry***:ab,ti,kw OR **worries***:ab,ti,kw OR **panic***:ab,ti,kw OR **phobi***:ab,ti,kw OR **'post-traumatic stress'**:ab,ti,kw OR **'posttraumatic stress*'**:ab,ti,kw OR **agoraphobi***:ab,ti,kw OR **'obsessive compulsive disorder*'**:ab,ti,kw OR **ocd**:ab,ti,kw OR **'psychological stress*'**:ab,ti,kw OR **'psychological distress*'**:ab,ti,kw OR **'emotional stress*'**:ab,ti,kw OR **'emotional distress*'**:ab,ti,kw OR **psychotrauma***:ab,ti,kw OR **'mental stress*'**:ab,ti,kw OR **'mental distress*'**:ab,ti,kw OR **ptsd**:ab,ti,kw OR **'traumatic stress*'**:ab,ti,kw OR **'traumatic distress*'**:ab,ti,kw OR **burnout***:ab,ti,kw OR **'burn-out*'**:ab,ti,kw OR **'work stress*'**:ab,ti,kw OR **'occupational stress*'**:ab,ti,kw OR **'compassion fatigue*'**:ab,ti,kw | **716,359** |
| **#2** | **'sudden cardiac death*'**:ti,kw OR **'sudden cardiac arrest*'**:ti,kw | **11,695** |
| **#1** | **'cardiovascular mortality'**/exp AND **'heart arrhythmia'**/exp OR **'heart arrest'**/exp OR **'sudden cardiac arrest*'**:ab,ti,kw OR **'sudden cardiac death*'**:ab,ti,kw OR **'karoshi death*'**:ab,ti,kw OR **'cardiac death'**:ab,ti,kw OR **'cardiac mortalit*'**:ab,ti,kw OR **'sudden death*'**:ab,ti,kw OR **'heart arrest*'**:ab,ti,kw OR **'heart arrhythmia*'**:ab,ti,kw OR **'heart ventricular arrhythmia*'**:ab,ti,kw OR **'heart ventricular tachycardia*'**:ab,ti,kw OR **'sudden heart death*'**:ab,ti,kw OR **'cardiac arrest*'**:ab,ti,kw | **190,108** |

**APA Psychinfo (EBSCO) 21-5-2021**

| **#** | **Query** | **Limiters/Expanders** | **Results** |
| --- | --- | --- | --- |
| S10 | S9 | Limiters – Academic journals | 501 |
| S9 | S5 OR S6 OR S8 | Search modes - Boolean/Phrase | 546 |
| S8 | S1 AND S3 | Narrow by Methodology: - retrospective study/ prospective study/ followup study  / longitudinal study  Search modes - Boolean/Phrase | 310 |
| S7 | S1 AND S3 | Search modes - Boolean/Phrase | 2,071 |
| S6 | S1 AND S3 AND S4 | Search modes - Boolean/Phrase | 391 |
| S5 | S2 AND S3 | Search modes - Boolean/Phrase | 28 |
| S4 | TI(Cohort OR longitudinal OR prospective* OR “follow-up stud*” OR “case-control stud*” OR “retrospective stud*” OR “cross-sectional stud*”) OR AB(Cohort OR longitudinal OR prospective* OR “follow-up stud*” OR “case-control stud*” OR “retrospective stud*” OR “cross-sectional stud*”) OR KW(Cohort OR longitudinal OR prospective* OR “follow-up stud*” OR “case-control stud*” OR “retrospective stud*” OR “cross-sectional stud*”) | Search modes - Boolean/Phrase | 287,679 |
| S3 | DE "Hoarding Behavior" OR DE "Generalized Anxiety Disorder" OR DE "Obsessive Compulsive Disorder" OR DE "Panic Attack" OR DE "Panic Disorder" OR DE "Separation Anxiety Disorder" OR DE "Anxiety Disorders" OR DE "Agoraphobia" OR DE "Claustrophobia" OR DE "Social Phobia" OR DE "Phobias" OR DE "Chronic Stress" OR DE "Perceived Stress" OR DE "Posttraumatic Stress" OR DE "Psychological Stress" OR DE "Social Stress" OR DE "Anxiety" OR DE "Occupational Stress" OR DE "Compassion Fatigue" OR TI(anxiet* OR anxious* OR worry* OR worries* OR Panic* OR phobi* OR “post-traumatic stress” OR “posttraumatic stress*” OR agoraphobi* OR “obsessive compulsive disorder*” OR OCD OR “psychological stress*” OR “psychological distress*” OR “emotional stress*” OR “emotional distress*” OR psychotrauma* OR “Mental stress*” OR “mental distress*” OR PTSD OR “traumatic stress*” OR “traumatic distress*” OR burnout* OR "burn-out*" OR "work stress*" OR "occupational stress*" OR “compassion fatigue*”) OR AB(anxiet* OR anxious* OR worry* OR worries* OR Panic* OR phobi* OR “post-traumatic stress” OR “posttraumatic stress*” OR agoraphobi* OR “obsessive compulsive disorder*” OR OCD OR “psychological stress*” OR “psychological distress*” OR “emotional stress*” OR “emotional distress*” OR psychotrauma* OR “Mental stress*” OR “mental distress*” OR PTSD OR “traumatic stress*” OR “traumatic distress*” OR burnout* OR "burn-out*" OR "work stress*" OR "occupational stress*" OR “compassion fatigue*”) OR KW(anxiet* OR anxious* OR worry* OR worries* OR Panic* OR phobi* OR “post-traumatic stress” OR “posttraumatic stress*” OR agoraphobi* OR “obsessive compulsive disorder*” OR OCD OR “psychological stress*” OR “psychological distress*” OR “emotional stress*” OR “emotional distress*” OR psychotrauma* OR “Mental stress*” OR “mental distress*” OR PTSD OR “traumatic stress*” OR “traumatic distress*” OR burnout* OR "burn-out*" OR "work stress*" OR "occupational stress*" OR “compassion fatigue*”) | Search modes - Boolean/Phrase | 362,907 |
| S2 | TI("sudden cardiac death*" OR “sudden cardiac arrest*”) OR KW("sudden cardiac death*" OR “sudden cardiac arrest*”) | Search modes - Boolean/Phrase | 171 |
| S1 | DE "Sudden Death" OR DE "Heart Disorders" OR ((DE "Arrhythmias (Heart)" OR DE "Bradycardia" OR DE "Fibrillation (Heart)" OR DE "Tachycardia") AND DE "Death and Dying")) OR TI("sudden cardiac arrest*" OR "sudden cardiac death*" OR “Karoshi Death*” OR “cardiac death” OR “cardiac mortalit*” OR “sudden death*” OR “heart arrest*” OR “heart arrhythmia*” OR “heart ventricular arrhythmia*” OR “heart ventricular tachycardia*” OR “sudden heart death*” OR “cardiac arrest*”) OR AB("sudden cardiac arrest*" OR "sudden cardiac death*" OR “Karoshi Death*” OR “cardiac death” OR “cardiac mortalit*” OR “sudden death*” OR “heart arrest*” OR “heart arrhythmia*” OR “heart ventricular arrhythmia*” OR “heart ventricular tachycardia*” OR “sudden heart death*” OR “cardiac arrest*”) OR KW("sudden cardiac arrest*" OR "sudden cardiac death*" OR “Karoshi Death*” OR “cardiac death” OR “cardiac mortalit*” OR “sudden death*” OR “heart arrest*” OR “heart arrhythmia*” OR “heart ventricular arrhythmia*” OR “heart ventricular tachycardia*” OR “sudden heart death*” OR “cardiac arrest*”) | Search modes - Boolean/Phrase | 12,064 |
