# Supplementary material for: Anxiety, Mental Stress, and Sudden Cardiac Arrest: Epidemiology, Possible Mechanisms and Future Research
Source: Front Psychiatry. 2022 Feb 3;12:813518. doi: 10.3389/fpsyt.2021.813518 (PMC8850954; doi:10.3389/fpsyt.2021.813518)
Supplement: Supplementary file 2 [file Data_Sheet_2.docx]

**Supplement 2. Table Anxiety**

**Supplemental table 1A. Anxiety related to sudden cardiac death in physically healthy samples**

| **Author, year** | **Design** | **N** | **Population** | **Patient characteristics** | | **Predictor** | **Outcome** | **Findings** |
| --- | --- | --- | --- | --- | --- | --- | --- | --- |
|  |  |  |  | **Male, n (%)** | **Age, in years range (M; SD)** |  |  |  |
| Albert et al. 2005 | Prospective cohort study | 71162^a^ | Nurses’ Health Study; registered nurses without CVD or cancer | 0 (0) | 42-67 (M=54) | CCEI phobic anxiety subscale in quartiles based on scores 0,1(ref)/2/3/4 or more | Incident SCD over 12 years follow-up (n=97) | Anxiety was associated with higher risk of SCD (p-trend adjusted for age: p=0.008; high vs low anxiety RR=1.77 (1.08-2.89).When adjusted for CHD risk factors smoking status, BMI, alcohol intake, menopausal status and postmenopausal hormone use, parental history of myocardial infarction before age 60 years, vigorous to moderate activity, usual aspirin use, valium use, p-trend=0.03; high vs low RR=1.59 (0.97-2.60); additionally adjusted for history of hypertension, history of hypercholesterolemia, history of diabetes, p-trend=0.06; high vs low RR=1.45, 0.88-2.38). |
| Kawachi et al. 1994a | Case-control design nested within a prospective cohort study | 1895^b^ | Normative Aging Study; community-dwelling veterans without chronic medical conditions; controls remained free of CHD | 1895 (100) | 21-80 (M=45, SD=9 in CHD cases, M=41, SD=9 in controls). | Scale of five items of the CMI with scores  0(ref)/1/2 or more, validated against CCI. | Incident SCD over 32 years follow-up (n=26). | Anxiety was associated with higher odds of SCD, although not always at a statistically significant level (age-adjusted associations: OR=2.66, 95%CI: 0.97-7.23 for men who scored 1, n=5; OR=5.73, 95%CI: 1.26-26.1 for men who scored 2 or more, n=2. Associations additionally adjusted for smoking, blood pressure, serum cholesterol, family history of heart disease and 2 or more drinks of alcohol per day: OR=2.96; 95%CI; 1.02-8.55 for men who scored 1; OR=4.46; 95%CI: 0.92-21.6 for men who scored 2 or more). Authors state that assessment of anxiety was made only at baseline and that average time to SCD was 17.5 (SD=8.3) years and that it may be possible that a proportion of subjects with normal anxiety scores at baseline developed higher scores later on. |
| Kawachi et al. 1994b | Prospective cohort study | 33858^c^ | Health Professionals Follow-up Study; male health professionals free of diagnosed CVD at baseline | 33838 (100) | 42-77 | CCEI phobic anxiety subscale categories based on scores 0,1(ref)/2/3/4 or more | Incident SCD over 2 years follow-up (n=16) | Anxiety was associated with higher risk of SCD (p-trend adjusted for age: p=0.002; RRs of SCD were 3.24, 95%CI 0.69-15.26 for men who scored 4 or more; 8.01, 95%CI 3.07-20.93 for men who scored 3, and 1.70, 95%CI 0.34-8.49 for men who scored 2). Additionally adjusted for smoking, alcohol intake, physical activity, body mass index, history of hypertension, diabetes mellitus, hypercholesterolemia, and family history of MI before the age of 60 years, p-trend was 0.01; highest vs lowest anxiety RR= 3.11, 95% CI = 0.58, 16.55. |

CCEI = Crown-Crisp Experiential Index; CHD = coronary heart disease; CMI = Cornell Medical Index; CVD = cardiovascular disease; SCD = sudden cardiac death. ^a^Cases with CHD outcomes other than sudden cardiac death (n=1197; non-fatal MI, non-sudden CHD death) were excluded for this systematic review. ^b^Cases with CHD outcome other than sudden cardiac death (n=376; non-fatal MI, angina pectoris, non-sudden CHD death) were excluded for this systematic review. ^c^ eCases with CHD outcome other than sudden cardiac death (n=152. non-fatal MI, non-sudden CHD death) were excluded for this systematic review.

**Supplemental table 1B. Anxiety related to sudden cardiac arrest or sudden cardiac death in samples at risk**

| **Author, year** | **Design** | **N** | **Population** | **Patient characteristics** | | **Predictor** | **Outcome** | **Findings** |
| --- | --- | --- | --- | --- | --- | --- | --- | --- |
|  |  |  |  | **Male, n (%)** | **Age, in years range (M; SD)** |  |  |  |
| Li et al. 2020 | Retrospective cohort study | 26204 | Patients with a principal diagnosis of acute MI in the National Inpatient Sample 2016. | 5975 (45.6) without anxiety, 5748 (43.9) with anxiety | (M=66; SD=14) | pre existing anxiety disorders (ICD-10 codes F064, F40-F409, F41-F419) | Incident SCA (ICD-10 codes I46-I469; n=641) | After propensity score matching for the patient and hospital demographics and relevant comorbidities, the anxiety group had a lower incidence of in-hospital cardiac arrest (2.1% vs 2.8%, p< .001) than the non-anxiety group. This protective effect was driven by the subgroup with non-ST segment elevation MI (2.3% vs 3.5%, p<001; 5.6% vs 5.7%, p=0.90 in STEMI subgroup). |
| Habibovic et al. 2013 | Prospective cohort study | 1012 | Patients implanted with a first-time ICD between May 2003 and November 2009 in three hospitals in The Netherlands; two datasets (including MIDAS) | 807 (80) | (M=61; SD=11) | STAI-S at the time of ICD insertion. Scores per tertile: 20-32/ 33-44/45-80. | aborted SCA: incident ventricular arrhythmia, including both antitachycardia pacing episodes and shocks over 1 year follow-up (n=194) | Anxiety at the time of ICD insertion was related to ventricular arrhythmias over 1 year follow-up in ICD patients (HR=1.02, 95%CI 1.00-1.03, p=0.01). This association remained significant after adjustment for age, sex, ICD-indication, angiotensin-converting enzyme, cardiac resynchronization therapy, diabetes mellitus, beta blockers and ACE-inhibitors (HR=1.02, 95%CI 1.01-1.03, p=0.01). High levels of anxiety were significantly associated with ventricular arrhythmias (highest vs. lowest tertile; univariate HR=1.84; 95% CI = 1.28-2.64; p=.001; multivariate HR=1.91; 95% CI = 1.33-2.75; p<.001). No significant anxiety by sex interaction was found. |
| Watkins et al. 2010 | Prospective cohort study | 947 | VAGUS Study; CHD patients with progressive, unstable or stable angina, hospitalized for coronary angiography | 658 (69) | 29-90 (M=62) | CCEI phobic anxiety subscale at baseline, 0-4 days post-catheterization in quartiles. Full sample: scores 0-1/2/3-4/5-16.  Men: 0/1-2/3-4/5-13.  Women: 0-1/2-3/4-6/7-16. | Incident SCD over 3 years follow-up (n=35) | Anxiety was unrelated to increased risk of SCD in the full sample (unadjusted HR=1.11; 95% CI, 0.81–1.52, p=.52; highest vs lowest quartile, n=23 vs 19), however, there was a significant gender-anxiety interaction: in men no significant relationship was found (unadjusted HR=0.86; 95% CI, 0.57–1.30, p=.48, highest vs lowest quartile, n= 9 vs 16), whereas in women, anxiety was associated with SCD (unadjusted HR=2.02; 95% CI 1.16–3.52, p=.01 highest vs lowest quartile, n= 14 vs 3; p-trend: unadjusted=.002; adjusted for age, LVEF, medical comorbidity =.007; additionally adjusted for beta-blocker use, antidepressant/anti-anxiety medication use, presence of an ICD, p=.015). |
| Frasure-Smith et al. 1995 | Prospective cohort study | 222 | Patients admitted to the Montreal Heart Institute who met at least two of three research criteria for an acute MI (i.e., typical chest pain, elevated cardiac enzymes, new Q waves on the ECG). | 173 (78) | 24-88 (M=60) | STAI-S at baseline, 5-15days post-MI; high anxiety ≥40 | SCD/SCA: arrhythmic events over first post-MI year (n=12) | Anxiety was not significantly associated with incident arrhythmic events during 1 year post MI (high anxiety in 3/12 arrhythmic events vs 36/168 no cardiac events; OR 1.21; 95%CI 0.30-4.83, p=0.79). Noteworthy, none of the 49 women, but 12 of the 173 men had an arrhythmic event. |

CCEI = Crown-Crisp Experiential Index; CHD = coronary heart disease; ECG = electrocardiogram; ICD = implantable cardioverter defibrillator; LVEF = left ventricular ejection fraction; SCA = sudden cardiac arrest; SCD = sudden cardiac death; STAI-S = state portion of Spielberger's State-Trait Anxiety Inventory.
