# Supplementary material for: Anxiety, Mental Stress, and Sudden Cardiac Arrest: Epidemiology, Possible Mechanisms and Future Research
Source: Front Psychiatry. 2022 Feb 3;12:813518. doi: 10.3389/fpsyt.2021.813518 (PMC8850954; doi:10.3389/fpsyt.2021.813518)
Supplement: Supplementary file 3 [file Data_Sheet_3.docx]

**Supplement 3. Table public events**

**Supplemental Table 2. Public events* related to sudden cardiac arrest or sudden cardiac death**

| **Author, year** | **Design** | **Exposure period** | **Control period** | **Outcome** | **Findings** |
| --- | --- | --- | --- | --- | --- |
| *Humanitarian disasters* | | | | | |
| Marijon et al. 2020 | cross-sectional database study of the Paris-SDEC registry | first 6 weeks of the Paris Covid-19 Lockdown, (March/April 2020) | corresponding weeks in 2012 to 2019. | All cases of sudden out-of-hospital cardiac arrest (SCA) in Paris and sub-urbs Hauts-de-Seine, Seine-Saint-Denis, Val-de-Marne (6.8 million inhabitants), occurring among adults, included since May 15, 2011 | During the pandemic period, the maximal weekly incidence of SCA per million inhabitants (weeks 13 and 14) was significantly higher than the maximal weekly incidence during the same period between 2012 and 2019 (26.64 versus 13.42, p<0·0001), with a rapid return to normal in the final weeks of the pandemic period. |
| Niiyama et al. 2014 | cross-sectional database study of the  Ministry of Health and Welfare in Japan | 8 weeks before, 4 weeks after and 5-40 weeks after the 2011 Great East Japan earthquake and tsunami on March 11 | corresponding periods in 2009 and 2010 | residents who died of sudden cardiac and unexpected death  in 12 municipalities of the  Iwate prefecture in northeast  Japan; 272 440 adult inhabitants in 2011. (ICD-10 codes I20 to I25, I46, I49.0, I50, I71, R96, and R98). | Compared with the previous years’ rate, the incidence (per 10 000  person-years) of SCUD for the initial 4 weeks after the disaster (acute phase) was double (33.5 vs 18.9), and thereafter the rate  returned to the previous level (standardized incidence rate = 1.71, 95% CI 1.33 to 2.16). Incidence prior to the disaster did not differ from control periods either. |
| Aoki et al. 2012 | cross-sectional database study of ambulance transport records in the Miyagi Prefecture | from 4 weeks before to 16 weeks after the Great East Japan earthquake on March 11 | corresponding periods in 2008, 2009 and 2010 | weekly occurrences of cardiac pulmonary arrest (or HF, ACS, stroke, pneumonia) in the Miyagi Prefecture. | Although the rate of definitive diagnosis at admission in the emergency rooms was comparable among the 4 years studied, the number of ambulance transports peaked on day 2 (March 12) followed by a gradual decline. For cardiopulmonary arrest, the Poisson regression coefficient of the week following the earthquake was 0.64, p <0.001. |
| Gold et al. 2007 | cross-sectional database study of the records of The Washington state Department of Health | the 48 hour and one-week periods after the following disaster events: Nisqually earthquake (28 February 2001) and after the ‘9-11’ terrorist attack in 2001. | the 48-hour and one-week periods for the one, two, and three weeks prior to the disaster events, as well as the corresponding 48-hour and one-week periods in 1999 and 2000, matched to the day of the week. | SCD in American men and women deceased in four counties in Washington: Thurston, Pierce, King, and Snohomish for Nisqually earthquake; or in King County, the most populous county in Washington for terrorist attack; 1.8 million people (ICD-10 codes 100-1002,105-109,111-128, or 133-151) | During the 48 hours following the Nisqually earthquake, 32 SCDs occurred in the affected four counties, compared to an average of 22 (SD=3.5) during the control periods, constituting a 45% increase in incidence (p = 0.02). During the exposure period of one week after the Nisqually earthquake, no significant differences in SCD were found as compared with the control period, nor during the 48 hours or one-week periods after the 2001 terrorist attack as compared with the corresponding control periods. |
| Leor et al. 1996 | cross-sectional database study of the records of the Department of the Coroner of Los Angeles County | the day of the earthquake and six days after the Northridge earthquake (17 January 1994) | the seven-day period before the earthquake and corresponding periods in 1991, 1992, and 1993 | SCD in Los Angeles County according to the definition of the Framingham Heart study. | On the day of the Northridge earthquake, there were 24 cases of SCD (z=4.41, p<0.001), which is a rise as compared with the control periods. There was a non-significant decline in the number of SCDs on the six days after as compared with the week before the earthquake (z=1.73, p=0.084). |
| *Important football tournaments* | | | | | |
| Simon et al. 2020 | cross-sectional database study; Polish Central Statistics Office of the Polish National Health Fund | three football tournament periods (2012 and 2016 European Championships, 2018 World Cup) | corresponding periods in five years before and after the tournaments. | SCA (ICD-10 code I46; n=6068) in ‘more than 10 million’ Polish men older than 35 years admitted to a hospital or an ambulatory care center in Poland; other ACE outcomes also reported. | There were no significant differences in SCA incidence between the combined exposure and control periods: 1.64 SCA per 100000 men in the exposure periods as compared with 1.51 SCA per 100000 men in the control periods; RR=1.08, 95% CI 0.87–1.35. |
| Niederseer et al. 2013 | cross-sectional database study; Bavarian Council for Statistics and Data management | days in the tournaments  period of the football World Cup 2006 on which the German national team played | reference periods in 2005 and 2006 | SCA (ICD-10 code I46; n=unknown) in Bavarian men and women (catchment area n=unknown) admitted to any of the 375 hospitals in Bavaria; other ACE outcomes also reported. | There were no significant differences between SCA hospital admissions during football World Cup 2006 and reference periods (mean number of SCA per day was 3.6 (SD=2.6) versus 3.0 (SD=1.6) during reference periods; IRR=1.21, 95%CI 0.61-1.81, p=0.34). There was no significant difference in sex distribution between exposure period and reference periods. |
| Katz et al. 2006 | cross-sectional database study; Mobile Intensive Care Units Registers | during the FIFA World Cup 2002 | the same period in 2001 | SCD (n=100) in Swiss men and women in a catchment area of 1.5 million inhabitants | The incidence of SCD increased with 63% (p=0.02) during the FIFA competition as compared with the control period, and this held for both men and women. |

ACE = acute cardiac/cardiovascular events; ACS = acute coronary syndrome; HF = heart failure; SCA = sudden cardiac arrest; SCD = sudden cardiac death

*disaster events or football spectatorship during important football tournaments as proxies for experienced emotional stress.
