# Supplementary material for: Anxiety, Mental Stress, and Sudden Cardiac Arrest: Epidemiology, Possible Mechanisms and Future Research
Source: Front Psychiatry. 2022 Feb 3;12:813518. doi: 10.3389/fpsyt.2021.813518 (PMC8850954; doi:10.3389/fpsyt.2021.813518)
Supplement: Supplementary file 4 [file Data_Sheet_4.docx]

**Supplement 4. Table live events**

**Supplemental table 3. Life events related to sudden cardiac arrest or sudden cardiac death**

| **Author, year** | **Design** | **N** | **Population** | **Patient characteristics** | | **Primary outcome measure** | **Findings** |
| --- | --- | --- | --- | --- | --- | --- | --- |
|  |  |  |  | **Male**  **n (%)** | **Age range or M (SD) in years** |  |  |
| Chang Liu et al. 2021 | retrospective case-control study | 36+36 | Cases were children and adults older than the age of 11 years who presented with SCA derived from the Cardiac Arrest Survivors with Preserved Ejection fraction Registry (CASPER). Controls were age- and sex-matched and recruited through advertisement via flyers, mailing list, website and Facebook | 36 (50) | M=47 (SD=15) | LCU of potentially stressful life events each with an assigned, differentially weighted numerical value based on the SRRS for non-adults, SSS, or RLCQ. The stressful life events were retrospectively assessed for occurrence during the year before the SCA (survivor group) or the same period as matched participants (control group), | No significant differences between SCA survivor group and age- and sex-matched control group with respect to LCU (M=252 vs 248; p>0.05). |
| Jeong et al 2018 | retrospective case-control  study | 95+95 | Cases were patients registered in the Cardiac Arrest Pursuit Trial with Unique Registration and Epidemiologic Surveillance (CAPTURES) project. Controls were unintentionally injured patients discharged from emergency department, age- and sex-matched, (NEDIS database). | 138 (73) | median = 69 | Major Life Events questionnaire (self-constructed), to retrospectively assess the experienced number of a list of 9 major life events experienced in the year before the SCA or the same period in the control group, obtained by a family member who lived with the participant. | A significantly higher number of major life events were found in the SCA survivor group as compared with the age- and sex-matched control group (58 versus 33 events; p>0.05). |
| Wicks et al. 2012 | retrospective case-crossover study | 490 | Married men and women who suffered SCA between 1988 and 2005, residents of King County, WA. | 397 (81) | 29-74; M=59 | Spouses were interviewed regarding the occurrence and estimated timing of 7 major life events during the year before the SCA. For every participant, the 1-month before SCA was compared with the 2–6-month period before SCA. | The occurrence of a major life event was higher in the month before the SCA than in the control period (OR=1.6; 95% CI = 1.1-2.4). |
| Cottington et al.  1980 | retrospective case-control study | 81+81 | Cases were Caucasian women, aged 25-64 years in Allegheny County, Pennsylvania who died suddenly from arteriosclerotic heart disease in a period of 1.5 years. Controls lived in the neighborhood of the cases and were matched on race, sex and age. | 0 (0) | Cases: M=54.5 (SD=8.3)  Controls: M=53.7  (SD=9.2) | Spouses (or the next of kin) of the cases and controls were interviewed regarding the occurrence of 28 major life events, selected from the Schedule of Recent Experience, during the six months before the SCD (cases) or interview (controls). The life-events were classified into three categories: positive, negative or unclassifiable. | Cases did not significantly differ from controls in total number of life-events (M=1.5 vs M=2.0 in controls; p>0.05), number of negative life-events (M=0.7 vs M=0.8 in controls; p>0.05) and number of unclassifiable life-events (M=0.6 vs M=0.7 in controls; p>0.05), but cases had experienced less positive life-events (M=0.2 vs M=0.5 in controls; p<0.001) and more frequently the death of a significant other (no means provided; p<0.01). |
| Rahe & Lind 1971 | retrospective case-crossover study | 39 | Cases were native Swedish men living in the greater Stockholm area, who had a sudden out-of-hospital death during the 3-month interval of October to December 1968. Cases were subdivided based on either or not reported signs and/or symptoms of CHD prior to their sudden deaths. Data were based on police and medical records. | 39 (100) | 38-70 (M=67) | LCU of potentially stressful life events each with an assigned, differentially weighted numerical value based on the Schedule of Recent Events questionnaire were filled out by wives, or occasionally relatives or close friends of the SCD cases. The stressful life events were retrospectively assessed for occurrence over the 3-4 years prior to death. For every participant, the 6-month period in the year before SCD was compared with the same period in the 2^nd^ and 3^rd^ year before SCD. | The final 6-months LCU were higher than those in the 2^nd^ and 3^rd^ year before SCD. This was true both for SCD cases with prior CHD histories (M=108 vs M=51^2yr^ vs M=59^3yr^; p-values <0.005; n=29) and those without prior CHD histories (M=169 vs M=51^2yr^ vs M=40^3yr^, p-values <0.01; n=10). No significant differences were found in LCU based on prior CHD histories either. |

CHD = coronary heart disease; LCU = life change units; RLCQ= Recent Life Changes Questionnaire; SCA = sudden cardiac arrest; SCD = sudden cardiac death; SRRS = Social Readjustment Rating Scale; SSS = Student Stress Scale.
